# Testicular blood supply is altered in the 41,XX^Y^* Klinefelter syndrome mouse model

**DOI:** 10.1038/s41598-020-71377-0

**Published:** 2020-09-01

**Authors:** Joachim Wistuba, Cristin Beumer, Ann-Sophie Warmeling, Reinhild Sandhowe-Klaverkamp, Jörg Stypmann, Michael Kuhlmann, Richard Holtmeier, Oliver S. Damm, Frank Tüttelmann, Jörg Gromoll

**Affiliations:** 1 Institute of Reproductive and Regenerative Biology, Centre of Reproductive Medicine and Andrology, University of Münster, University Clinics, Albert-Schweitzer-Campus 1, Building D11, 48149 Munster, Germany; 2 Department of Cardiovascular Medicine, University of Münster, Albert-Schweitzer-Campus 1 Building A1, 48149 Munster, Germany; 3 European Institute for Molecular Imaging (EIMI), University of Münster, Waldeyerstraße 15, 48149 Munster, Germany; 4 Institute of Human Genetics, University of Münster, Vesaliusweg 12-14, 48149 Munster, Germany

**Keywords:** Physiology, Diseases, Endocrinology

## Abstract

Hypergonadotropic hypogonadism is a major feature of Klinefelter syndrome (KS), assumed to be caused by testicular hormone resistance. It was previously shown that intratesticular testosterone levels in vivo and Leydig cell function in vitro seem to be normal indicating other functional constraints. We hypothesized that impaired testicular vascularization/blood flow could be a co-factor to the observed hypergonadotropic hypogonadism. We evaluated the testicular vascular system by measuring blood vessel sizes during postnatal development and testis blood flow in adult 41,XX^Y^* mice. Proportional distribution and size of blood vessels were analyzed during testicular development (1, 3, 5, 7, 10, 21 dpp*,* 15 wpp). While ratios of the vessel/testis area were different at 15 wpp only, a lower number of smaller and mid-sized blood vessels were detected in adult KS mice. For testicular blood flow determination we applied contrast enhanced ultrasound. Floating and reperfusion time for testicular blood flow was increased in 41,XX^Y^* mice (floating: XY* 28.8 ± 1.69 s vs XX^Y^* 44.6 ± 5.6 s, p = 0.0192; reperfusion XY* 19.7 ± 2.8 s vs XX^Y^*: 29.9 ± 6.2 s, p = 0.0134), indicating a diminished blood supply. Our data strengthen the concept that an impaired vascularization either in conjunction or as a result of altered KS testicular architecture contributes to hormone resistance.

## Introduction

The presence of one or more supernumerary X-chromosomes causes Klinefelter syndrome (KS, 47,XXY) in men. This chromosomal aneuploidy occurs at a high incidence of 1–2 in 1,000 male births and is the most frequent genetic cause for male infertility. Characteristic for KS is the hypergonadotropic hypogonadism characterized by highly elevated gonadotropins and low to very-low testosterone (T) levels resulting from testicular hormone resistance. Germ cell loss prevails, generally leading to azoospermia in nearly all KS patients^1–5^. During the last years, co-morbidities have been associated with KS such as cardiovascular disease (shortened QTc times), increased risk for pulmonary embolism and peripheral vascular diseases^6–11^. As for vascular problems, reduced diameters of brachial, common carotid and femoral as well as of the abdominal arteries have been reported.

Low T levels in KS patients have numerous effects on health and are likely contributing to the majority of symptoms but can be clinically treated by T substitution positively influencing some but not all of the symptoms^4,12,13^. However, and more problematic, androgen replacement interferes with the endocrine feedback by down-regulating Follicle Stimulating Hormone (FSH) and Luteinizing Hormone (LH). This negatively impacts the sperm retrieval success in KS patients with remaining focal spermatogenesis^14–16^. Consequently, it is of crucial importance to elucidate the underlying mechanisms causing hypergonadotropic hypogonadism to potentially develop novel therapeutic options for the treatment of KS patients.

Previously, disturbed Leydig cell function was thought to be causative for serum T deficiency. However, it was shown experimentally that Leydig cells are hyperactivated in the 41,XX^Y*^ mouse model with an significant increased mRNA expression of all Leydig cell markers analyzed and that LH receptor signaling per se is fully functional. Thus, other mechanisms, besides of a Leydig cell failure seem to be involved^17^. This assumption was further strengthened when intratesticular testosterone (ITT) values were found to be comparable between 41,XX^Y^* and 40,XY* littermates, a finding which was later confirmed in a KS patient cohort^17,18^. Consequently, although the endocrine feedback regulation is definitely disturbed, impaired T production cannot be the reason for the lowered peripheral serum T levels. Testes of KS patients are phenotypically described as small and firm with volume of approximately 2–2.5 mL vs. the healthy normal range above 12 mL per testis^19,20^ which is due to a dramatically altered testicular architecture caused by germ cell loss and Leydig cell hyperplasia. We reported previously that the vascularization of testes in 30 weeks old mice was altered mainly due to a changed blood vessel composition^18^. The fact that Leydig cell function is seemingly normal and the blood vessel composition is changed led us to speculate whether an impaired testicular vascularization could contribute to the phenotype of hypergonadotropic hypogonadism.

To address this hypothesis we utilized our 41,XX^Y^* mouse model to evaluate proportional vessel distribution during postnatal development of the testis and testicular blood flow in adult mice.

## Results

### Testicular vascularization during development

**Figure 1:**
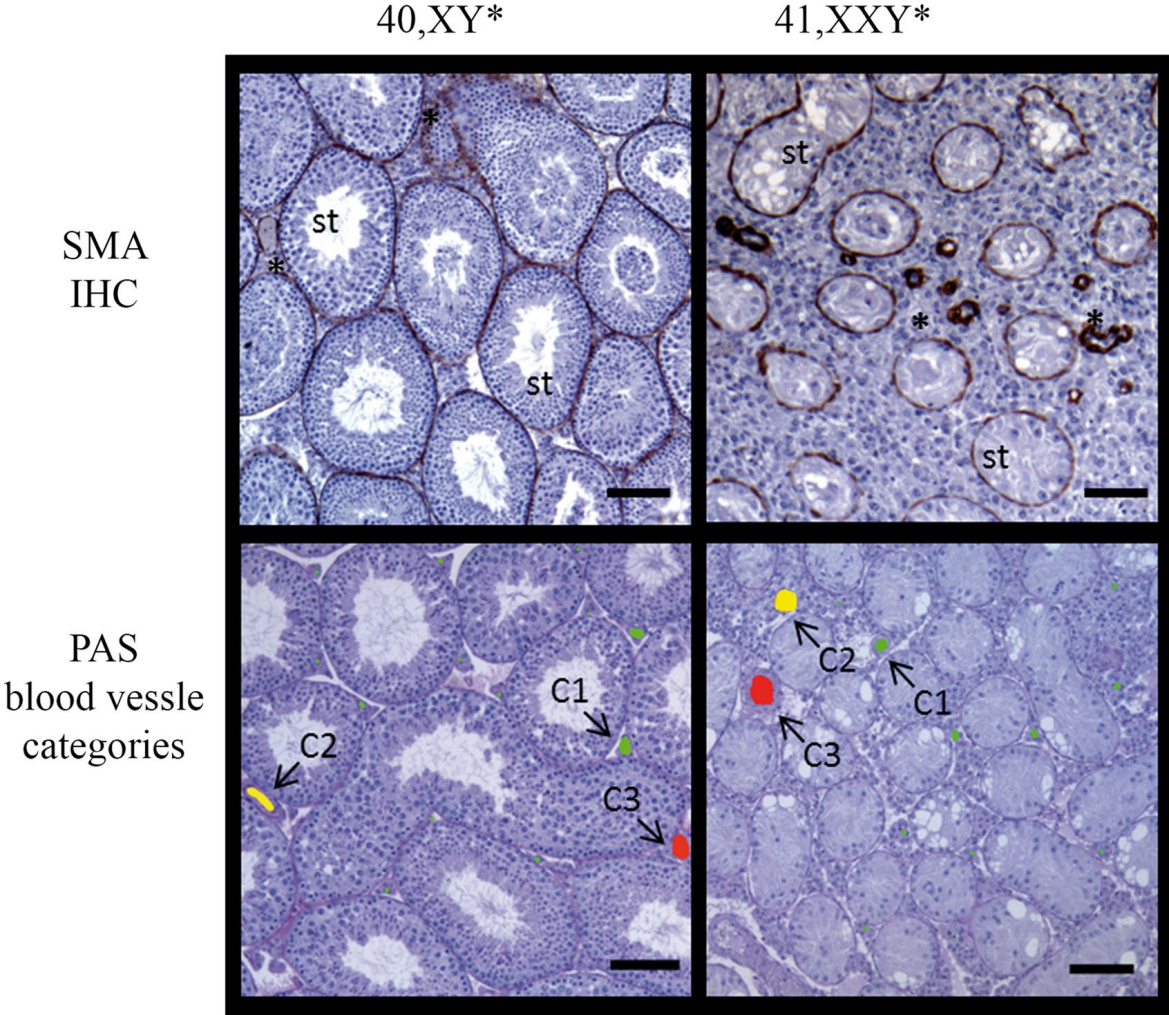
Vessel categories for vascularization analyses in 40 XY* and 41,XX^Y^* mice. Upper panel: testicular cross-sections stained for alpha-smooth muscle actin (SMA) to visualize blood vessels (stars) between the seminiferous tubules (st). Such sections were used for quantitative analyses. Lower panel: PAS-stained testicular cross-sections with vessels colored for illustration of size categories (< 80 µm^2^: green, 80–1,000 µm^2^: yellow, > 1,000 µm^2^g: red). Scale bar equals 200 µm.

**Figure 2:**
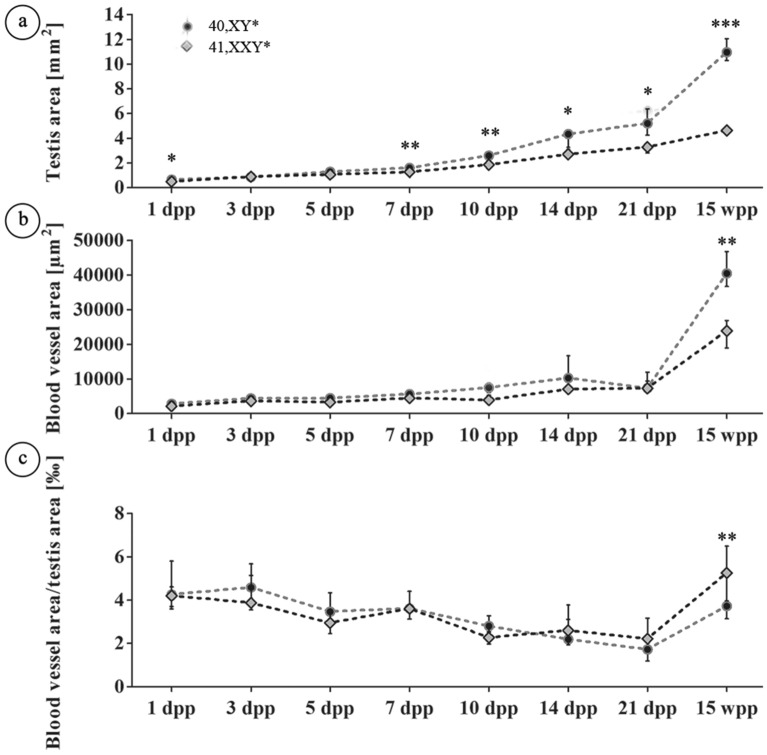
Analysis of testicular vascularization over the postnatal development (1, 3, 5, 7, 10, 14, 21 *dpp, 15 wpp*) in 40,XY* and 41,XX^Y*^ mice. Testicular cross sections stained immunohstochemically for SMA were analyzed for the total area covered by blood vessels (blood vessel area) and the whole cross sectional area (testis area) as described by Tüttelmann et al.^18^. (**a**) Testis area. (**b**) Blood vessel area. (**c**) Ratios of vessel area/testis area. In (**a**–**c**), medians with interquartile ranges are shown; *p < 0.05; **p < 0.01; ***p < 0.001.

Testicular vascularization was quantitatively analyzed using central testicular cross sections which underwent immunohistochemical staining for SMA, detecting both, seminiferous tubule walls by marking myoid peritubular cells as well as blood vessels. Thus this staining allowed to clearly distinguish seminiferous tubules from vessels by structural differences and enabled accurate evaluation.We examined testicular cross sections during postnatal development in neonatal (1 dpp), juvenile (3, 5, 7, 10 dpp), pubertal (14, 21 dpp) and adult (15 wpp) 41, XX^Y^* males and their corresponding 40 XY* littermates as controls. Areas of the entire testicular cross section and those covered by blood vessels and the ratios thereof were measured (Figs. 1, 2a–c). Testis size (area) steadily increased during development in mice of both karyotypes. Significant differences became evident at day 7, 10 and 14 dpp prior to puberty and at 21 dpp resembling the onset of puberty. Differences in testis area were most prominent at 15 wpp in fully adult mice when full spermatogenesis was ongoing in control mice but lacking in 41,XX^Y*^ mice (Fig. 2a). Testicular blood vessel’s areas were similar in both karyotypes up to 21 dpp, but highly significant differences were noted for time point 15 wpp for 41,XX^Y*^ mice showing a reduced blood vessel area growth (Fig. 2b).

Vascularization degree was normalized for the smaller testicular size of 41,XX^Y^* mice when calculating the ratios of the blood vessels area divided by absolute testis area analyzed (Fig. 2c). Although the degree of vascularization slightly decreased over the early phases of testicular development, no significant changes were noticed until 21 dpp. At 15 wpp, in young adult mice the ratio was significantly higher in 41, XX^Y^* testes, although the amount of total blood vessels was smaller. This is likely due to the much bigger testis area in 40,XY* males in which spermatogenesis is running at a maximum (Fig. 2a,c).

### Testicular vascularization according to blood vessels size categories in adult males

**Figure 3:**
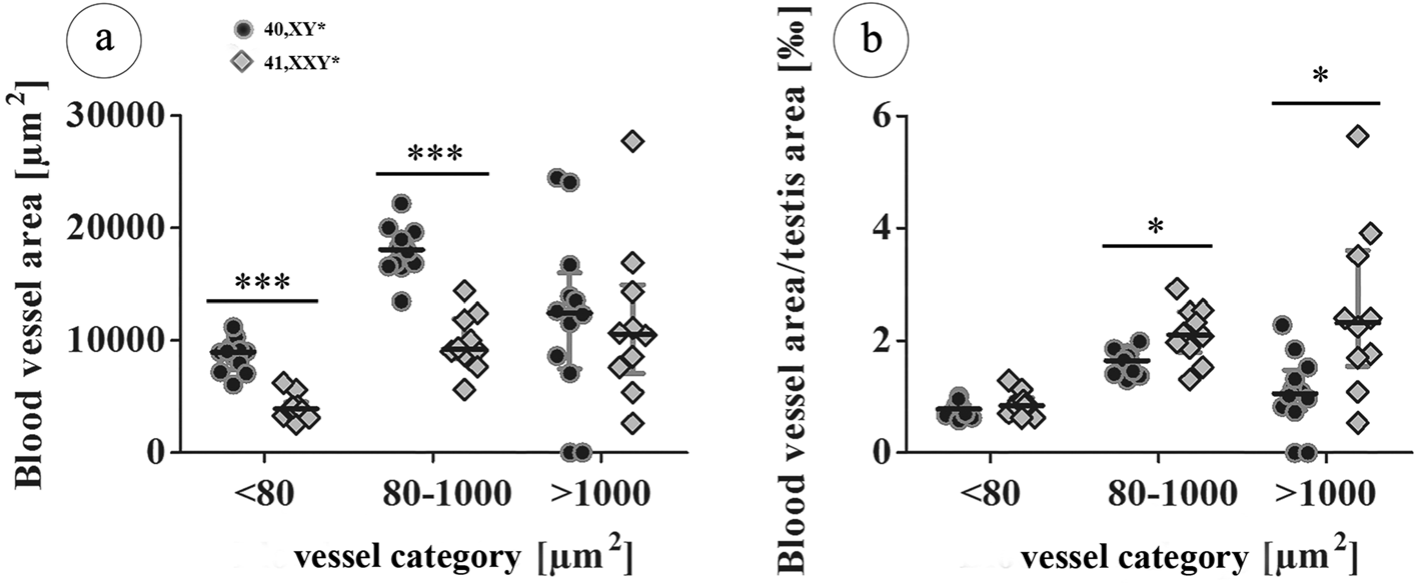
Vessel size distribution in testes of 40,XY* and 41,XX^Y*^ mice. Blood vessels were grouped according to the three categories < 80 µm^2^, 80–1,000 µm^2^ and > 1,000 µm^2^. The blood vessel area/testis area ratio was determined considering always only vessels of the respective size categories. (**a**) Area covered by vessels assigned to the three size categories in 15 wpp testes. (**b**) Blood vessel area/testis area ratio considering vessels of three size categories in 15 wpp testes. All data are presented as median values with interquartile ranges. Dot plots additionally include the individual values obtained from each testis analyzed; *p < 0.05; **p < 0.01; ***p < 0.001.

Using a three-category system for blood vessels, we noticed that the areas of the smallest (< 80 µm^2^) vessels belonging to category 1 and the intermediate (80–1,000 µm^2^) vessels of category 2 were significantly larger in 40,XY* testes (p = 0.0001 respectively). No significant differences between the karyotypes regarding the area covered by the largest vessels (> 1,000 µm^2^) representing category 3 could be detected, Fig. 3a). For each size category, the respective blood vessel area/testis area ratio was determined, indicating the testicular degree of vascularization (Fig. 3b). The degree of vascularization regarding the smallest blood vessels did not reveal any differences, whereas the degree of vascularization for category 2 and 3 was significantly increased in 41,XX^Y*^ mice (p = 0.0111).

### Determination of testicular blood flow by contrast enhanced ultrasound analysis (CEUS)

**Figure 4:**
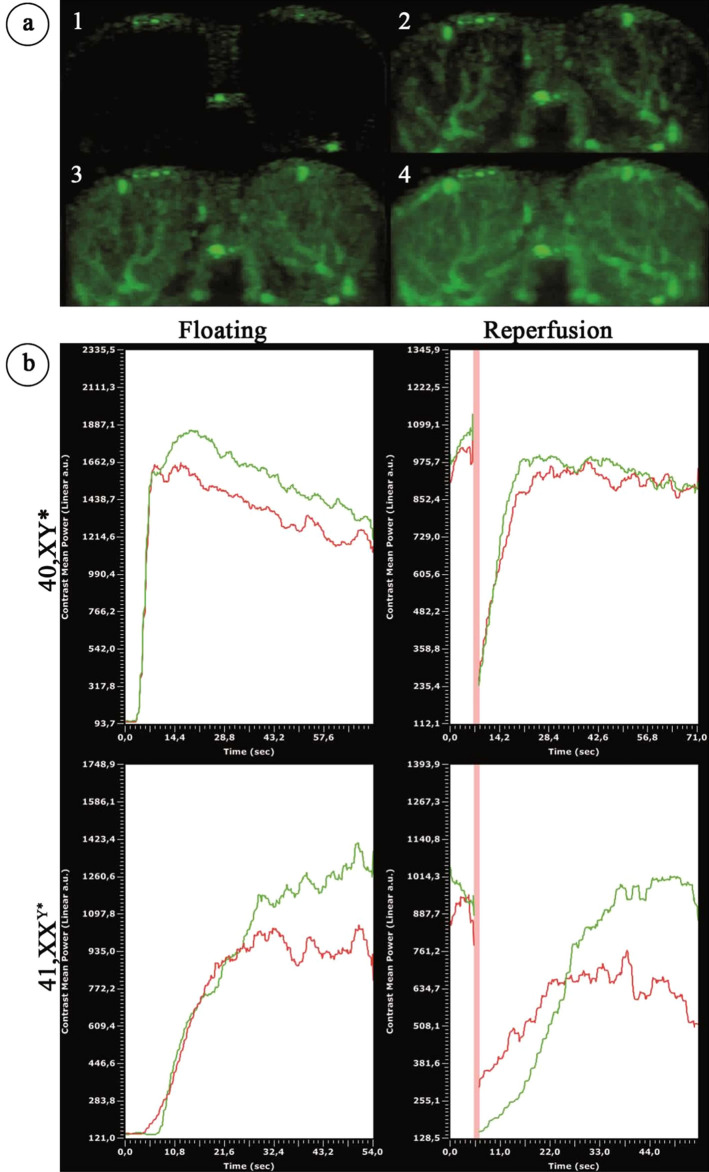
Contrast-enhanced ultrasound analysis of testicular blood flow in adult 40,XY* and 41,XX^Y*^ mice. (**a**) Perfusion of the testes by contrast agent: after injection before the contrast agent reached the testis (1). Contrast agent appears in the testicular area (2) and the covered area increases (3) until the state of maximal filling after perfusion: “floating” is reached (4). (**b**) Left upper and lower graph: graphical illustration of blood flow by ultrasound signal intensity in 40,XY* and 41,XX^Y*^ mice. Right upper and lower graph: after floating, the contrast agent signal was destroyed by a boost (high power ultrasound pulse) before the testes was flooded again with contrast agent (“reperfusion”). Floating as well as reperfusion duration (“time to peak”) served as the functional readout for the testicular blood flow. Green = signal measured in right; red in left testis. The red vertical line reflects the boost.

**Figure 5:**
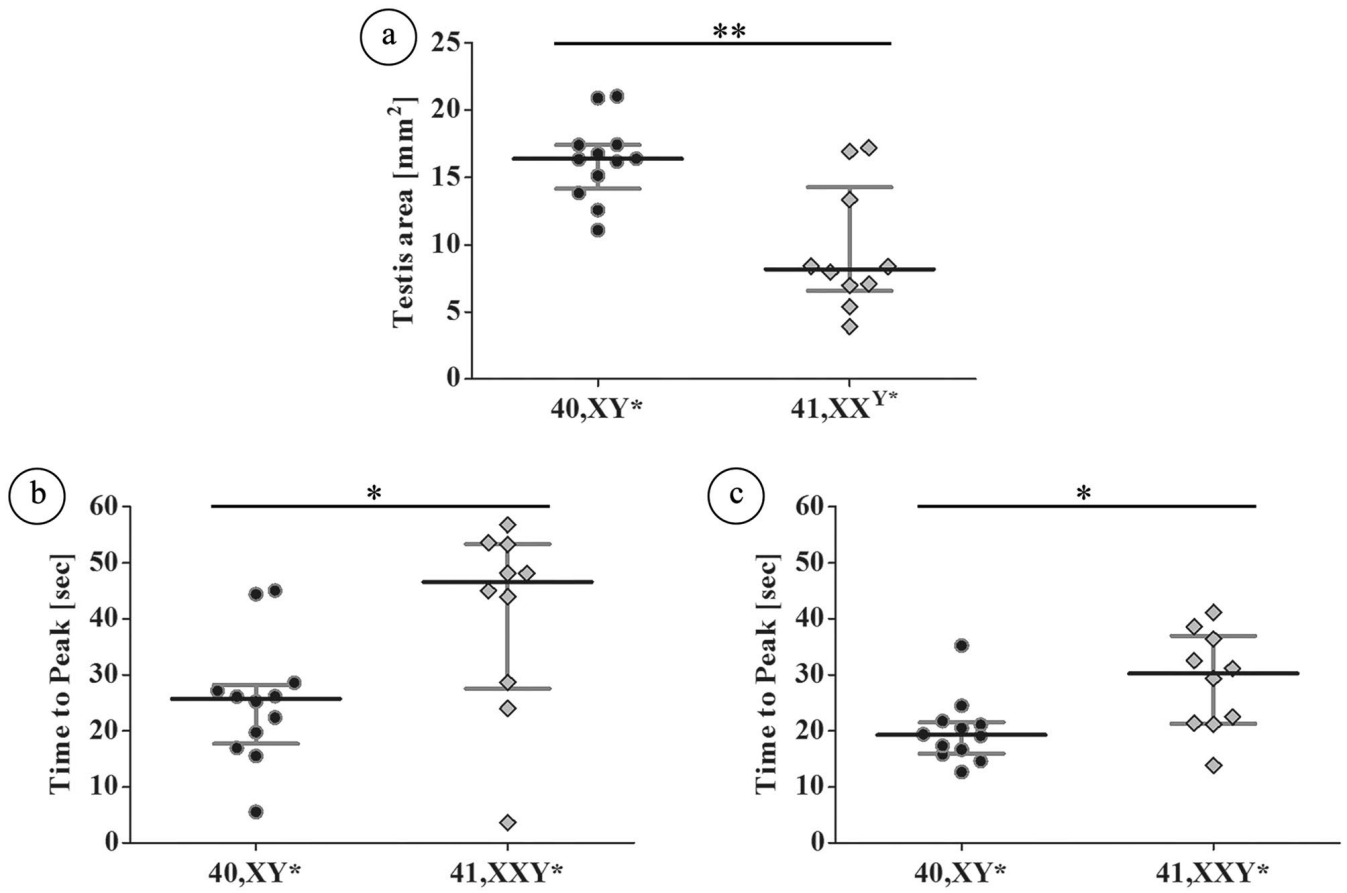
Quantitative analysis of the testicular blood flow in adult (15–16 wpp) 40,XY* and 41,XX^Y*^ mice by contrast-enhanced ultrasound. Testicular perfusion by contrast enhanced ultrasound: (**a**) testicular area reflecting the different testis sizes between both groups; (**b**) time to peak of floating was significantly lower in wild type males; (**c**) time to peak of reperfusion was also significantly lower in wild type males. In both measurements the increased time for testicular perfusion in XX^Y^* mice indicates hampered blood flow. Black dots: 40,XY*, grey diamonds: 41,XX^Y^*. *p < 0.05; **p < 0.01.

Taking advantage of a technology which enables to visualize and measure blood flow in testis in vivo, we addressed the question whether the noticed changes in the blood vessels architecture in 41,XX^Y*^ mice also effects blood flow in the testis (Fig. 4). Contrast enhanced ultrasound analysis revealed significantly smaller testes in 41,XX^Y^* mice compared to 40,XY* controls (p = 0.0062, XX^Y^* 4.6 ± 0.10mm^2^; XY* 11.1 ± 0.34mm^2^, Fig. 4a). For blood flow measurements, the perfusion of the testes was videotaped. One major difference was immediately obvious between both groups (Supplementary Video S1) since floating of testes of 41,XX^Y^*mice was remarkably delayed (Supplementary Video S1, Fig. 5).

Using CEUS we then measured the time needed to float and to reperfuse the testes. Time to peak of floating (p = 0.0192) and reperfusion of testes was significantly extended in XX^Y^* compared to XY* mice. Floating and reperfusion time for testicular blood flow was significantly increased in 41,XX^Y^* mice (floating: XY* 28.8 ± 1.69 s vs XX^Y^* 44.6 + 5.6 p = 0.0192; reperfusion XY* 19.7 ± 2.8 s vs XX^Y^*: 29.9 + 6.2p = 0.0134), indicating a diminished capacity (Figs. 4b, 5a–c, Supplementary Video S1).

### Serum testosterone evaluation

Serum testosterone was measured in 15 week old mice used in the contrast enhanced ultrasound experiments (XY*: n = 12; XX^Y^*: n = 10). Serum T levels of 41,XX^Y^* males (4.808 ± 0.9 nmol/L) were significantly lower than in 40 XY* males (14.40 ± 3.7 nmol/L).

## Discussion

By using quantitative methods and sophisticated in vivo measurements of the blood flow, this study addresses a mechanism potentially underlying the testicular hormone resistance, which resembles a hallmark of the KS phenotype. We recently reported that LC function and ITT levels are nearly in the normal range in testes of KS patients and in 41,XX^Y^*mice giving room for the idea that a Leydig cell impairment is not causative for the observed low peripheral T levels. Thus, a different mechanism must be acting on the disturbed endocrine feedback.

When analyzing the testicular vascularization during development, our finding that at 15 weeks of age the area covered by blood vessels related to the entire testis area was significantly larger in 41,XX^Y^* mice seemingly contradicts our previous observations from 30 week old animals^18^. In mice of this age, we found that the absolute area covered by blood vessels but also the blood vessel to testes area ratio were smaller. This observation might point to a progressive degenerative process, in which the depletion of germ cells, which is complete during the first postnatal weeks^17,21^, affects testicular vascularization in the long-term. The nearly complete lack of germ cells leads to compaction of the testis, which eventually evokes compensatory processes (i.e. additional bigger vessels are formed) possibly responding to the emptied spermatogonial niches. An alternative explanation could be that vessels are still there (resulting in a higher vessel/testis ratio because of the relatively smaller testis size) for a while but get increasingly lost because of the changing testicular architecture, a phenomenon which is also seen in other mouse models losing the germ line^22^. The fact that we see these changes only at 30-weekold animals points to an age-dependent process. It might also well be that at a more advanced age hyalinization of tubules and tubular ghosts, well known to be present in testis of KS patients, are the result of these vascular changes.

Using the CEUS system, we were able to monitor blood flow in vivo to get deeper insights on the effects of the changed vascularization. As we found a significantly increased time to peak values for both, testicular floating as well as reperfusion, we proved that the observed altered vascular situation results in significantly hampered blood flow in the XX^Y^* testes. It is tempting to assume that the impaired perfusion we observed in 41,XX^Y^* males is caused by the lack of smaller vessels which prevent a proper distribution of blood within the testis. This phenomenon is prone to worsen at a higher age when the larger vessels are reduced in addition^18^. It might also well be, that progressive testis degeneration and tubular hyalinization are additionally enhanced by a decreasing blood supply, i.e. that a “vicious circle” accelerates the damage of the testicular structures with time, an observation also made in patients with KS^23^.

The disturbed testicular circulation may be one co-factor to the endocrine phenotype associated with KS. Only little is known on a putative transport system, which enables gonadotropins, which represent large protein hormones, to pass the blood/testis barrier to interact with their target cell, namely the Sertoli and Leydig cells. It appears plausible that the loss of capillaries in 41,XX^Y^* mice interferes with this and results in an insufficient stimulation of LC in the presence of highly elevated LH serum levels. In line with this are studies from Belli et al.^24^ who further stimulated KS patients with human Chorionic Gonadotropin (hCG) and could evoke an increase in T biosynthesis. However, alternatively, also T release could be hampered, which is supported by the notion that T is produced in sufficient amounts in the testis of KS patients^18^.

Future approaches might prove or disprove this concept experimentally by testing the release of T from testis organoids closely resembling the in vivo situation to elucidate whether testis compactness plays a role. Alternatively, LH function could be studied, e.g. through the administration of small diffusible LHR agonists^24,25^ to address the accessibility of LH to the Leydig cells. This approach could clarify the issue to which extent LH action can be significantly increased in the testis.

One of the most prominent and best known features of KS is the progressive loss of germ cells during development, likely to start already pre- to perinatally^21,26^. As a previous study indicated that the localization of SSCs and interstitial cells is related to the testicular vasculature and proposed that the formation of the vascular network precedes and impacts germ line niche establishment^27^, altered vascularization might be involved in these processes or at least they are linked to each other. Two scenarios seem conceivable: the predisposed angiogenetic reduction of the testicular vascularization might provoke the loss of germ cells. Another potential explanation is that the germ cells are depleted due to further undefined causes resulting in an altered testicular architecture that induces a secondary reduction of vascularization. Which of both hypotheses—if any—reflects the actual situation, needs to be investigated in more detail in future studies. In both cases germ cell loss and altered testicular vasculature would impact each other, possibly elucidating a common cause for altered endocrine regulation and germ cell depletion in KS.

It remains to be solved whether X-chromosomal genes escaping from X-inactivation are involved in one or both processes and whether these changes are a secondary consequence of other features of this chromosomal disease. The gene *KDM6A*/*Kdm6a*, which escapes X chromosomal inactivation in both, human and this mouse model has been shown previously, is known to play a “master switch” role in early organogenesis of the cardiovascular system suggesting a genetic influence on the changes observed in the vascular system in KS^28,29^.

Our findings were obtained in a mouse model and although these findings appear to offer a plausible explanation for the hypogonadal status of KS, a confirmation that this observation also holds true in patients remains to be shown. If so, therapeutic approaches aiming at improving testicular vascularization are envisaged.

Taken together, our results for the vasculature architecture in the testes of male 41,XX^Y^* mice point to a grossly changed vessel composition, including an altered contribution of vessels from the different categories. This ultimately leads to a changed testicular blood flow. Both factors together might play a role for the well-known testicular hormone resistance and germ cell loss in KS.

## Materials and methods

### Animals

Male 41,XX^Y^* mice as well as their 40,XY* littermate controls, were obtained from the institutes breeding colony of the B6Ei.Lt-Y* strain^26^. Animals were kept in macrolon type II cages on a layer of wood shavings. The cages were enriched with paper towels as well as red macrolon nest boxes. Animals had ad libitum access to pelletized food (Altromin 1324, Altromin, Lage, Germany) and tap water. Further keeping conditions were a 12:12 LD cycle at a constant room temperature of 24 °C and a humidity of 50%. All applications and protocols were performed in accordance with the national and European legislation for animal care and experiments and were approved by the animal ethics committee of the Landesamt für Natur, Umwelt und Verbraucherschutz Northrhine Wesfalia (regional authorithy for animal ethics LANUV NRW; License numbers: 84–02.05.20.13.115 and 84–02.05.30.12.084). Karyotypes were determined along two different routes using established in house methods. The karyotype of immature mice was determined by Xist-RT-PCR according to a protocol from Werler et al*.*^30^. Briefly, RNA was isolated from fresh liver tissue by Ultraspec (AMS Biotechnology, Wiesbaden, Germany). RNA (1 lg) was transcribed into cDNA using random Hexamer Primer and Reverse Transcriptase Superscript II (Invitrogen, Darmstadt, Germany). The 83-bp-long Xist fragment was amplified by reverse transcriptase PCR with 1.5 lL cDNA (mouse primer: forward: 5¢-GAGCCCAAAGGGACAAACAA-3¢; reverse: 5¢AGTTCTGCTGAGATGTAAT-GTAGCTGTATAG-3¢; thermocycling conditions: 3 min 94 °C, 28 cycles: 94 °C: 15 s, 60 °C: 30 s, 72 °C: 1 min; final extension: at 72 °C, 5 min). In contrast, adult mice were karyotyped for presence of sex chromosomes using Fluorescence in Situ Hybridisation of 200–300 µl blood samples. In short, 750 µl Biocoll (Biochrom AG, Berlin, Germany) were used for separation before cells were cracked with KCl and methanol/glacial acetic acid. Of every sample, 0.5 µl was dropped onto a slide and dried on a heating plate. The slides were denatured in 70% formamide 2 × SSC (saline sodium citrate buffer) at 68 °C and dehydrated through an ethanol series. Probes (1200 XMCY3-02; 1189-YMF-02; Cambio, Cambridge, UK) were incubated overnight. Afterwards the slides were placed in 50% formamide 2 × SSC at 43 °C for 15 min, before being transferred into heated 2 × SSC for 10 min and then for another 10 min at room temperature. The slides were counterstained with Hoechst 33258 and mounted in Vectashield (Vector Laboratories, Burlingame, USA)^17,31^. Numbers of animals used in the single experiments are reported in the respective paragraphs.

### Contrast enhanced ultrasound analysis (CEUS)

Mice (XX^Y^*: n = 10, XY* n = 12) aged 15–16 weeks were initially anaesthetized by inhalation of 4.5% isoflurane/l O_2_ in a narcosis box (Landmark VSA-2100, Vetland Medical, Louisville, USA) before 1.5% isoflurane/l O_2_ was applied continuously during the measurements inside the Vevo imaging station (VisualSonics Inc., Toronto, Canada). Non-targeted contrast agent Vevo MicroMarker™ (VisulaSonics Inc.) solved isotonic sodium chloride (0.9%, B. Braun, Melsungen, Germany) was injected via the tail vein using a catheter (Vevo^®^ MicroMarker™ TVA Cannulation Kit, VisualSonics) and an ointment (Bepanthen^®^, Bayer AG, Leverkusen, Germany) was applied to prevent eye dehydration. The animal was fixed onto the handling table connecting the paws to electrodes of the monitoring unit using contact gel (Aquasonic^®^ 100, Parker Laboratories, Inc., Fairfield, USA). Animal’s heart rate, an electrocardiogram (ECG), body temperature, respiration rate and blood pressure were monitored. The testicular region was shaved to avoid confounding signals, positioned by an elastic strap^32,33^, and de-gassed ultrasound gel (Aquasonic^®^ 100, Parker Laboratories, Inc., Fairfield, USA) was applied to the scrotum. The Micro Scan™ transducer (MS-250, VisulaSonics Inc.) was positioned in B-Mode allowing simultaneous observation of both testes with a focus depth of 7–8 mm. Analysis was performed in a contrast specific imaging mode in a two dimensional manner^32,34^. The ultrasound frequency was set to 18 MHz and applied for imaging at 10% of the maximum transmission power and for signal acquisition contrast gain was set to 32 dB to maintain a constant background signal in all measurements. Confounding by respiration was excluded by gating the acquisition between two respiration peaks. Recording started before injection of 45 µl of the agent (rate 15 µl/s) in order to avoid microbubble destruction. When both testes were filled with the contrast agent for the first time, duration of this process was measured; designated “floating”. After floating, the microbubbles present in the region of interest [ROI defined by tracing the ultrasound image; the area (mm^2^)] were destroyed by a single ultrasound pulse (“boost”), before testicular reperfusion by the circulating agent was determined, a process designated “reperfusion”. The period required (‘time to peak’) for reaching maximal signal intensity (maximum blood amount in the testis) served as a measure of testicular blood supply for floating and reperfusion.

### Histology

All adult animals were sacrificed by cervical dislocation before anaesthesia abated whilst immature animals were killed by immediate decapitation. Both testes were removed and fixed in Bouin’s solution for up to 24 h before being dehydrated, and embedded in paraffin. Multiple 5-µm sections of each sample were obtained. Every second slide was stained with periodic acid-Schiff (PAS)/hematoxylin^17^, and examined using the Olympus BX61 microscope with an attached Retiga 400 R camera (Olympus, Melville, NY, USA) and integrated CellSens imaging software (Olympus, Melville, NY, USA). The whole testis of immature animals and the central testicular part of adults was processed, serial sections generated, and the section with greatest diameter selected for subsequent evaluation of vascularization by immunostaining against a-smooth muscle actin (SMA, see below).

### Analysis of vascularization

The vascular composition in testicular tissues of 41,XX^Y*^ and 40,XY* littermate mice was histologically evaluated at 1 dpp (XX^Y^*: n = 5, XY* n = 7), 3 dpp (XX^Y^*: n = 5, XY* n = 5), 5 dpp (XX^Y^*: n = 5, XY* n = 6), 7 dpp (XX^Y^*: n = 5, XY* n = 6), 10 dpp (XX^Y^*: n = 5, XY* n = 5), 14 dpp (XX^Y^*: n = 5, XY* n = 5), 21 dpp (XX^Y^*: n = 5, XY* n = 6) dpp and 15–16 (XX^Y^*: n = 10, XY* n = 12) wpp*.* Developmental stages were chosen in consistence with previously published studies^17,21^. PAS stained cross sections taken from the center of each testis were analyzed microscopically for identification of the testicular region with the greatest circumference. Corresponding slides from this central region were then immunohistochemically stained against SMA for detection of the vessels and serial pictures covering the entire area were taken or, if required, single pictures were assembled and analyzed as previously published (magnification 100×; Olympus BX61, Adobe Photoshop CS3, Version 10.0 (Adobe Systems Incorporated, San Jose, USA^18^). To address testicular blood vessel composition during development we assigned the vessels to three categories. (1) smaller than 80 µm^2^, which resembles mainly capillaries; (2) larger than 80 µm^2^ but smaller than 1,000 µm^2^ indicative for intermediate and (3) larger than 1,000 µm^2^ for bigger venules and arterioles (Fig. 1). From the region of testis with the greatest diameter two sections per animal were evaluated in all developmental stages. Per developmental stage and karyotype at least 200 vessels were evaluated ranging from 32 to 102 vessels per animal.

### Immunohistochemical staining (IHC) for alpha smooth actin (SMA)

For detection and size measurement of blood vessels, IHC against SMA was performed according to a protocol previously published^35^. We used a primary antibody directed against smooth muscle actin (SMA; mouse-monoclonal anti-SMA, A2547, 1:1000, Sigma-Aldrich, Hamburg, Germany) which is encoded by the ACTA2 gene. Testicular sections treated with corresponding mouse (I5381) IgG antibodies served as control (1:1000, Sigma-Aldrich, Hamburg, Germany). After overnight incubation, sections were washed three times in TBS and the corresponding secondary antibody, conjugated to horse-radish peroxidase (chicken anti-mouse HRP, sc2954, 1:100, Santa Cruz Biotechnology, Inc. Heidelberg, Germany) were applied. Protein expression was visualized using 3,3′-diaminobenzidine as chromogen. Hematoxylin was applied as counterstain (Fig. 1). Samples were analyzed microscopically and documented as described above.

### Serum testosterone determination

Trunk blood was collected and measurement of serum T concentrations in adult (15 week) 40,XY and 41,XX^Y^* male mice from the enhanced ultrasound experiments were conducted utilizing a solid-phase double-antibody radioimmunoassay (RIA). The RIA was performed using iodinated tracer (testosterone-3-(*O*-carboxymethyl) oximino-2-[125I] iodohistamine, Amersham International, Germany), a rabbit testosterone-3 (carboxymethyloxime)-BSA antiserum and a secondary antibody against rabbit IgG (Bio-Rad Laboratories Incorporation, Hercules, USA). Recovery after ether extraction was monitored by addition of tracing amounts of [1β, 2β-3H] testosterone (NEN Life Sciences, Inc., Boston, MA, USA) to the sera and the results were corrected accordingly. Detection limit was 0.72 nmol/l. Duplicate serum samples from each animal were analyzed for serum T levels. The intra- and inter-assay coefficients of variation were 4.7 and 5.8%, respectively^36–38^.

### Statistics

Statistical analysis was performed using Microsoft Excel 2010 (Microsoft Corporation, Redmond, USA) and GraphPad Prism Version 5.0 (GraphPad Software, San Diego, USA). Due to group sizes and as missing normal distribution the nonparametric Mann Whitney U test was utilized for data analysis. For testosterone measurements, due to the small sample numbers, a Gaussian distribution could not be assumed and a regular two-way ANOVA and Bonferroni posttests were performed. Values were considered significantly different when p < 0.05. If not stated otherwise, medians are given.

## Supplementary information

Supplementary information 1

Supplementary information 2
